# Practical Views of Medical Education

**Published:** 1850-08

**Authors:** 


*Practical views of Medical Education.*—The undecided state of public opinion in regard to some of the fundamental points in a course of medical education, including among other things the portion of the term of pupilage proper to be spent in attendance on lectures, is thought by the undersigned, to justify a further consideration of the subject. In some of its relations, this subject has been already discussed, in the Transactions of the American Medical Association for 1849, in two reports, pages 353 and and 359, to which the reader is particularly referred. The following condensed, but more general view of the subject of medical education, is now respectfully submitted to the members of the Association.

*Boston, July* 10, 1850.

---

1. Medical instruction should be adapted to the power of students to receive and retain what is communicated to them, and should beconfined to what is important to them in subsequent life.

2. In modern times the constituent branches of medical science are so expanded, that they are not acquired by any physician in a life-time, and still less by a student during his pupilage. The same is true even of many individual branches. It is not, therefore, to be conceded that "a scheme of scientific instruction should embrace the whole science, and no part should be omitted;" nor that "a well-digested plan of lectures embraces all that is to be known and taught," Medical science has at this day become so unwieldly, and contains so much that is unnecessary, at least to beginners, that the attempt to explain to students the whole, is likely to involve the result of their learning but little.

3. In Chemistry, at the present time, a thorough adept is unknown. No man living knows all the recorded facts, or all that is to be known and taught in that science. Organic chemistry alone fills large volumes, though yet in its infancy.

4. In Materia Medica there are some thousands of substances and their compounds, which possess what is called a medicinal power. Yet it is not probable that any physician effectively reads the one half, or remembers one quarter, or employs in his yearly practice one tenth, of the contents of the common dispensatories.

5. In Pathology, so complicated and various are the conditions attendant on the individual forms of disease, and their relations with idiosyncracy, temporary condition and external agency, with organic lesions and functional disturbances, that few of the most experienced pathologists can be said to understand their whole science, or to be always competent to its successful application.

6. In Etiology, the theoretical literature of causes has spread itself out to an extent, which is burdensome and unprofitable. It is true, that "man, from his nature, is subject to suffering disease and death;"—but it is not equally apparent, that "the causes by which these conditions are produced, are ascertainable." We know nothing of the vehicle of cholera or influenza, nor is it probably in the power of any physician, by any art, or application of his knowledge, to produce in any common healthy man, a case of common pneumonia, or of acute rheumatism,—of diabetes or Bright's kidney,—of hypertrophy or of cancer,—or even of a common boil, or wart.

7. In Theraputics, many hundred volumes exist, such as would not have existed, could a knowledge of the cure of diseases be made so easily tangible, that it could be spread before the student in the three or five years of his pupilage.

8. In Anatomy, general and special, microscopic and transcendental;—in Physiology, with its intricate ramifications; —in Surgery, of which several subordinate specialities constitute distinct living professions; it is not to be admitted that the means or time of any ordinary course of lectures, can furnish full and complete instruction. Certainly it must be difficult to arrange a course of lectures on any of the extensive sciences which now constitute medicine, if it be indeed true, that "the teachers are not justifiable in suppressing any portion."

9. It is the business of lecturers in medical schools, to condense and abridge the science which they respectively teach, to distinguish their essential and elementary principles, to sift carefully the useful from the superfluous, and to confine the scope of their teachings, as far as possible, to what is true and profitable, and likely to be remembered and used by their hearers. It is unfortunately too true, that "in an extended system of instruction, there is much the student will not master, much that will have escaped his attention, much which he ought to know, that he has not learned." The remedy appears to be, to teach him well what he can and should master, and briefly to point out to him the sources, fortunately abundant, from which he may obtain the rest.

10. Much injury is done to the cause of true learning by medical assumption, amplification and exaggeration, by premature adoption of novelties, and tenacity of theories, personal or espoused. Students, in all former years, have spent much time in learning, what has afterwards cost them both time and trouble to unlearn ;—in acquiring, not merely the truths of science, but the crude announcements and plausible doctrines of sanguine or ingenious men. How much time has been wasted in our distinguished seminaries, in acquiring, the visionary, and now neglected, theories of Rush and Broussais !

11. The most commonly exaggerated branch of medical science is theraputics. Enlightened physicians well know, that many diseases are incurable, and that others are subject to laws of duration, which cannot be interrupted by art. Yet students often return from medical schools persuaded that their instructers know how to cure a large part of these diseases, and if others are less fortunate it is attributable to their own fault.

12 Medical teachers should keep pace with the progress of their respective sciences. Yet in their haste for the promulgation of novelties, they should not omit to give the proper consideration to the older and more settled principles of science. Medical men are liable to commit the error of adopting premature opinions, unsound practice and inconvenient changes of language aud nomenclature, some times for a love of display, and sometimes for a want of self reliance, and a fear of being thought behind the literature of their time.

13. The length of a course of lectures is not the measure of its value to the student. A course of lectures should not outlast the curiosity of its hearers, nor their average pecuniary ability to attend. Custom in this country has generally fixed the limit of these things at about four months. A comprehensive and judicious course, confined to the enforcing of necessary points, is far more profitable than a more discursive course to a wearied and diminishing audience.

14. Lectures are chiefly wanted to impress by demonstration the practical branches of science, and they are most effective in places where the facilities for such demonstrations can be commanded. Anatomy requires extensive exhibitions by the teacher, and personal dissections by the student. Chemistry and Materia Medica require illustrations by specimens and exprriments. Pathology needs the aid of autopsies, museums and the clinical demonstrations of large hospitals. A knowledge of Obstetrics is not perfected without apparatus and practice.* Surgery is acquired by witnessing numerous operations, surgical diseases, illustrated explanations, and by personal practice on the dead body. Physical exploration is wholly demonstrative. A knowledge of auscultation can no more be acquired from books, or abstract lectures, than a knowledge of music, or of individual physiognomy.

15. The intermediate period between lectures, should be spent by students in active and original study, approved and confirmed by regular recitations, and by such opportunities as can be commanded, for practical, personal experience. Private schools for small classes, and the private teachings of individuals, who are suitably qualified and situated, are more advantageous for two-thirds of the year, than either the fatiguing jostle of overcrowded rooms, or the listless routine kept up by the survivors of a passive class.

16. The usefulness of a medical school depends not so much on the length of its session, as upon the amount of education, preliminary and ultimate, which it requires, the fidelity with which it exacts its own professed requisitions, and the train of healthy exertion, active inquiry, and rigid, methodical, self-regulating study, to which it introduces its pupils. The longest lectures are of little use to students who want a common education, and whose medical education does not qualify them afterwards to observe, to inquire, and to discriminate. The exacted evidence of three years of well conducted study, is better than the exhibited ticket of a six months' course.

17. The subjects most important to be well taught in medical schools, are the elementary principles which constitute the frame-work of medical sciences, and the mode of thought and inquiry which leads to just reasoning upon them. After these, most attention should be given to selecting and enforcing such practical truths, as will most certainly be wanted by the young practitioner in his future career of responsibility.

18. The things to be avoided by medical teachers, are technicalities which are unintelligible to beginners,—gratuitous assumptions and citations

---

* To which we would add demonstrations with the living subject.—Editor Buffalo Medical Journal.

of doubtful authorities,—prolix dissertations on speculative topics,—excess-ive minuteness in regard to subjects which are intricate and but little used, and therefore destined to be speedily forgotten. To these may be added controversies, superfluous personal eulogiums and criminations, and all self-exaggeration, personal or local.

JACOB BIGELOW, *Prof of Materia Medica and Clinical Medicine.*
WALTER CHANNING, *Prof of Midwifery and Med. Jurisprudence.*
JOHN WARE, *Prof. of Theory and Practice of Medicine.*
JOHN B. S. JACKSON, *Prof of Pathological Anatomy.*
OLIVER W. HOLMES, *Prof. of Anatomy and Physiology.*
HENRY J. BIGELOW, *Prof. of Surgery.*
E. N. HORSFORD, *Prof. of Chemistry.*

